# The adoption and compliance to central line-associated bloodstream infection insertion and maintenance bundle programs in intensive care unit settings across Canada

**DOI:** 10.1017/ice.2024.189

**Published:** 2025-02

**Authors:** Zhi Lin Zhou, Anada Silva, Kristine Cannon, Blanda Chow, Jeannette L. Comeau, Chelsey Ellis, Charles Frenette, Amir Hadzic, Jennifer Happe, Lynn Johnston, Kevin C. Katz, Jamal Khan, Joanne M. Langley, Bonita E. Lee, Santina Lee, Marie-Astrid Lefebvre, Cassandra Lybeck, Allison McGeer, Andrew Neitzel, Jennifer Parsonage, Connie Patterson, Caroline Quach, Michelle Science, Stephanie W. Smith, Nisha Thampi, Reena Titoria, Jen Tomlinson, Joseph Vayalumkal, Kathryn N. Suh, Jocelyn A. Srigley, the Canadian Nosocomial Infection Surveillance Program

**Affiliations:** 1 Public Health Agency of Canada, Ottawa, ON, Canada; 2 Alberta Health Services, Calgary, AB, Canada; 3 IWK Health, Halifax, NS, Canada; 4 Dalhousie University, Halifax, NS, Canada; 5 The Moncton Hospital, Moncton, NB, Canada; 6 McGill University Health Centre, Montréal, QC, Canada; 7 Kelowna General Hospital, Kelowna, BC, Canada; 8 IPAC Canada, Edmonton, AB, Canada; 9 North York General Hospital, Toronto, ON, Canada; 10 Stollery Children’s Hospital, Edmonton, AB, Canada; 11 Department of Pediatrics and Child Health, University of Manitoba, Winnipeg, MB, Canada; 12 Montreal Children’s Hospital, Montréal, QC, Canada; 13 Sinai Health, Toronto, ON, Canada; 14 Centre Hospitalier Universitaire Sainte-Justine, Montréal, QC, Canada; 15 The Hospital for Sick Children, Toronto, ON, Canada; 16 Children’s Hospital of Eastern Ontario, Ottawa, ON, Canada; 17 Provincial Health Services Authority, Vancouver, BC, Canada; 18 Health Sciences Centre, Winnipeg, MB, Canada; 19 Alberta Children’s Hospital, Calgary, AB, Canada; 20 The Ottawa Hospital, Ottawa, ON, Canada; 21 BC Children’s & BC Women’s Hospitals, Vancouver, BC, Canada

## Abstract

Evidence-based insertion and maintenance bundles are effective in reducing the incidence of central line-associated bloodstream infections (CLABSI) in intensive care unit (ICU) settings. We studied the adoption and compliance of CLABSI prevention bundle programs and CLABSI rates in ICUs in a large network of acute care hospitals across Canada.

## Background/objectives

Central line-associated bloodstream infections (CLABSI) are a preventable patient safety concern in Canadian hospitals. Patients with CLABSI experience high morbidity and mortality, with 30-day all-cause mortality reported at 10.4%–31.6%, depending on the intensive care unit (ICU) setting.^1^ Evidence-based insertion and maintenance bundles have been effective in reducing the incidence of CLABSIs in ICU settings.^2^ The Canadian Patient Safety Institute (CPSI) bundle for CLABSI prevention was adopted in adult and pediatric hospitals starting in 2005 and has shown success in reducing CLABSI rates.^3^ The Children’s Hospitals’ Solutions for Patient Safety (SPS) bundle was adopted across the US and Canadian pediatric hospitals since 2013 and was found effective in reducing CLABSI rates.^4^ Although CLABSI prevention bundle programs are used within Canadian hospitals, information on national adoption and compliance with specific bundle components is limited. In this report, we studied the adoption and compliance of CLABSI insertion and maintenance bundle programs among hospital ICUs participating in the Canadian Nosocomial Infection Surveillance Program (CNISP). We also compared CLABSI rates between hospitals that did and did not adopt a CPSI or SPS bundle program.

## Methods

CNISP is a collaboration between the Public Health Agency of Canada, the Association of Medical Microbiology and Infectious Disease Canada, and sentinel hospitals that conduct national surveillance of healthcare-associated infections.^5^ At the time of the study, the CNISP network included 88 acute care hospitals and had reported quarterly data on CLABSI rates since 2009.^6^

We distributed an expert-reviewed, piloted, standardized electronic questionnaire to 88 CNISP hospitals from February 7 to March 31, 2023 (Supplemental Material). Participating hospitals self-reported information on the following items in one or more ICU settings (adult mixed, adult cardiovascular surgery (adult CV), pediatric (PICU), and neonatal (NICU)): (1) CPSI and SPS CLABSI prevention bundle program adoption^3,4^, (2) individual bundle component implementation, and (3) bundle compliance. Survey results were reported nationally and by region (Western: British Columbia, Alberta, Saskatchewan, and Manitoba; Central: Ontario and Québec; Eastern: Nova Scotia, New Brunswick, Prince Edward Island, and Newfoundland and Labrador; Northern: Nunavut). Survey results were then merged with CNISP CLABSI surveillance rate data collected from 2009 to 2022 using standardized national case definitions.^7^ We conducted descriptive analysis and calculated CLABSI incidence rate ratios (IRR) with 95% CI using median-unbiased estimations. All analyses were conducted in R 4.3.0.

## Results

Forty-six of 88 hospitals (52%) reported on 35 adult mixed ICUs, 13 adult CV ICUs, 16 NICUs, and 11 PICUs. The regional distribution of participating hospitals reflected the distribution of hospitals in the CNISP network, with most reporting hospitals located in central Canada (48%, n = 22/46), followed by western (33%, n = 15/46), eastern (17%, n = 8/46), and northern Canada (2%, n = 1/46). Of the 46 hospitals that responded to the survey, 31 (67%) reported adopting either CLABSI bundle program (CPSI or SPS). Hospitals that adopted a bundle program were more likely to be larger-sized, teaching hospitals in central Canada compared to those that did not (Supplemental Table). Bundle adoption in ICUs was highest in adult CV (77%, n = 10/13), followed by PICUs (73%, n = 8/11), adult mixed (66%, n = 23/35), and NICUs (56%, n = 9/16). For adult and pediatric/neonatal ICUs, the CPSI bundle program was adopted between 2006 and 2021 and 2008 and 2021, respectively, while pediatric/neonatal ICUs adopted the SPS bundle between 2015 and 2021.

**Figure 1.**
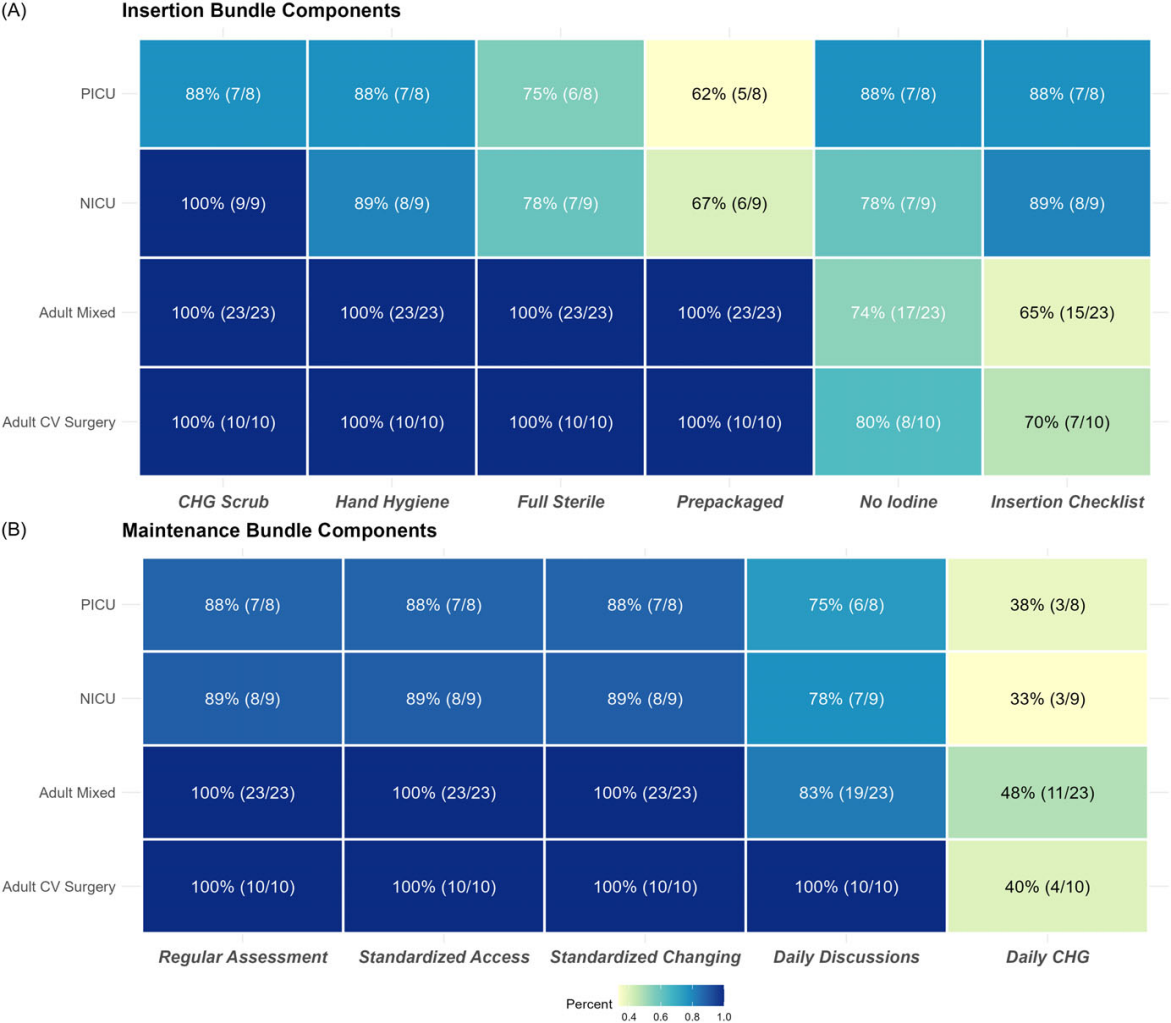
Central line-associated bloodstream infection prevention bundle insertion and maintenance component implementation (n = 31). Adult mixed, adult mixed patient intensive care unit; CLABSI, central line-associated bloodstream infection; CPSI, Canadian Patient Safety Institute; adult CV surgery, adult cardiovascular surgery intensive care unit; ICU, intensive care unit; NICU, neonatal intensive care unit; PICU, pediatric intensive care unit; SPS; Solutions for Patient Safety. *Note:* The bundle components listed are a combination of both CPSI and SPS bundles. A. Bundle insertion components include chlorhexidine scrub (If there is a contraindication to chlorhexidine, tincture of iodine, an iodophor, or 70% alcohol can be used as alternatives), hand hygiene, full sterile barrier for providers and patients, prepackaged or filled insertion cart, tray or box, no iodine ointment, and insertion checklist. B. Bundle maintenance components include regular assessment of dressing to assure clean/dry/occlusive, standardized access procedure, standardized dressing, cap and tubing change procedures/timing, daily discussion of line necessity, functionality and utilization including bedside and medical care team members, and daily chlorhexidine treatment (frequency of chlorhexidine treatments not specified in CPSI bundle).

Figure 1 displays the implementation of individual bundle components among CPSI or SPS participating sites by ICU setting. Across all ICUs, “CHG Scrub” was the most commonly implemented insertion bundle component (88%–100%), while “Insertion Checklist” was the lowest (65%–89%). Most maintenance bundle components were implemented across all ICUs except “Daily chlorhexidine (CHG) treatment” (33%–48%). Compared to pediatric/neonatal ICUs, adult ICUs consistently implemented more insertion (90% vs 82%) and maintenance bundle components (72% vs 63%).

Only 20%–30% of adult ICUs (adult CV, n = 2/10; adult mixed, n = 7/23) and 56%–62% of pediatric/neonatal ICUs (NICU, n = 5/9; PICU, n = 5/8) evaluated bundle compliance with a reported compliance of 90%–100% and 75%–100%, respectively.

**Figure 2.**
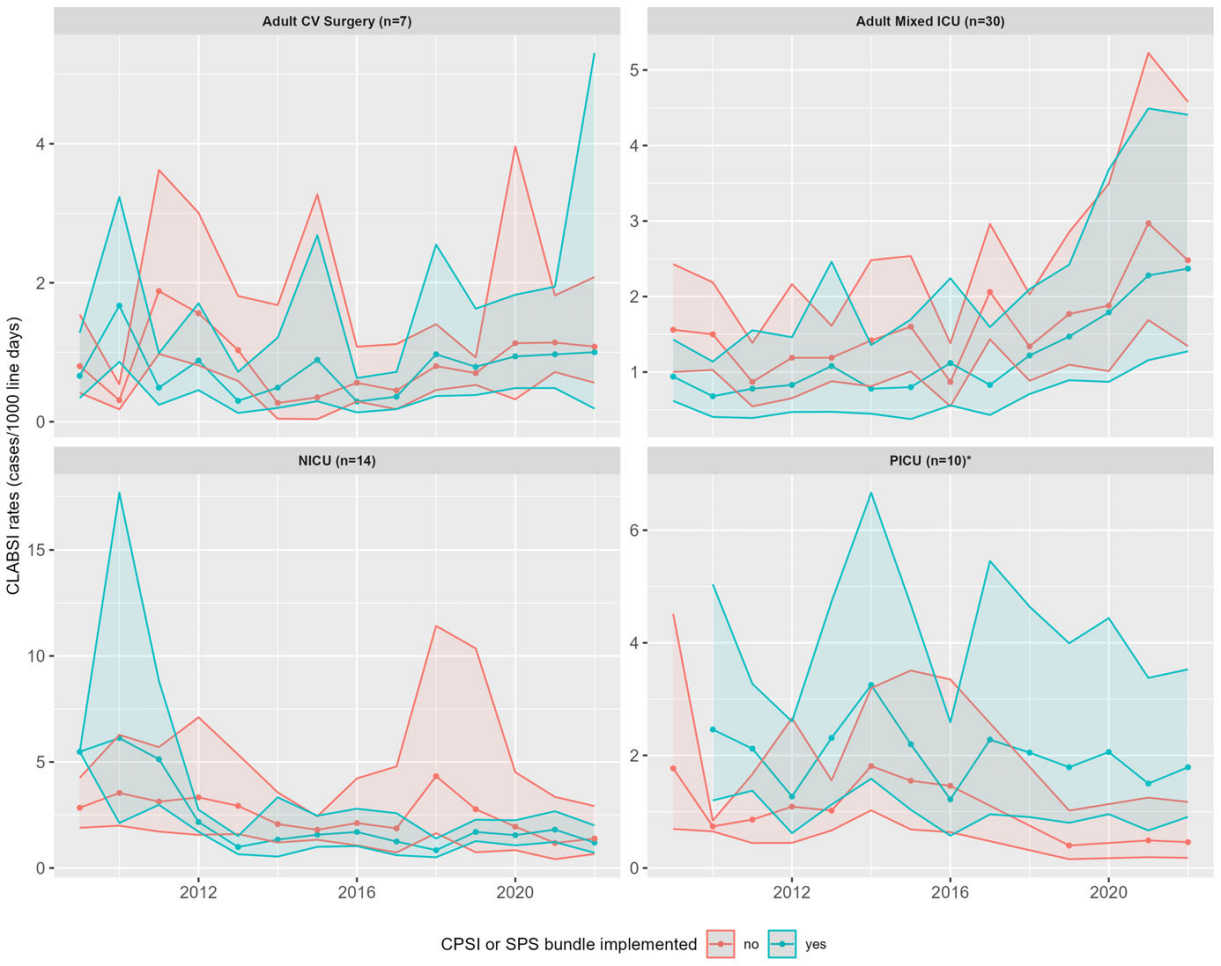
Central line-associated bloodstream infection rates across intensive care unit settings stratified by bundle implementation. Adult mixed, adult mixed patient intensive care unit; CLABSI, central line-associated bloodstream infection; CPSI, Canadian Patient Safety Institute; adult CV surgery, adult cardiovascular surgery intensive care unit; ICU, intensive care unit; NICU, neonatal intensive care unit; PICU, pediatric intensive care unit; SPS; Solutions for Patient Safety. *Note:* Only sites that participated in the survey and also submitted consistent CLABSI surveillance data were included in this figure. CLABSI rates were calculated by dividing the total count of CLABSI by the total number of line days for each group per year. Inclusion in the bundle group solely depended on whether any bundle was implemented in the ICU during that year. For example, if a site implemented a bundle in 2017, it would belong to the “no bundle” group until 2016 and then belong to the “yes bundle” group from 2017 onward. *Please interpret results with caution as only one site is present in the PICU “no bundles” group from 2016 onward.

Figure 2 compares CLABSI rates in ICUs with and without the adoption of a prevention bundle. From 2009 to 2022, CLABSI rates were significantly lower in adult mixed ICUs (IRR = 0.82; 95% CI, 0.75–0.90) and NICUs (IRR = 0.66; 95% CI, 0.58–0.75) that had a CLABSI prevention bundle program adopted compared to those without. Rates in PICUs (IRR = 1.57; 95% CI, 1.27–1.96) were higher among sites with either bundle adopted and similar regardless of bundle program adoption status in adult CV ICUs (IRR = 0.81; 95% CI, 0.63–1.05).

## Discussion

We evaluated the site-reported adoption of CLABSI prevention bundle programs and the implementation of individual bundle components in Canadian ICUs. Although the majority of participating hospitals adopted either the CPSI or SPS bundle in their ICUs, the implementation of specific bundle components varied by ICU. Our analysis showed the adoption of a CLABSI bundle program to be associated with lower CLABSI rates among select ICU settings. This finding is comparable to a study that found CLABSI prevention bundles statistically reduced CLASBI rates per 1,000 line days in adult mixed ICUs (IRR = 0.45; 95% CI, 0.38–0.52) and NICUs (IRR = 0.47; CI, 0.38–0.59).^2^

We evaluated the joint implementation of SPS and CPSI bundle components due to overlapping recommendations. “CHG Scrub” was most commonly implemented across all ICUs (88%–100%), which was consistent with findings from previous studies.^8^ In contrast, “Daily CHG” was the least implemented maintenance bundle component, also consistent with previous literature.^2,8^ Barriers to daily CHG treatments may be due to safety concerns related to skin integrity and the higher prevalence of CHG-resistant organisms.^9^ Hospitals participating in the CPSI bundle may have a low implementation of the “Insertion Checklist” insertion because the use of the checklist is not listed as a distinct component, but rather integrated in all insertion components in the bundle.

Overall, evaluation of bundle compliance ranged between 20% and 62%, similar to previous studies.^2,8^ Lower reported evaluation of bundle compliance could be due to the longstanding implementation of bundles across hospitals, resulting in compliance evaluation only during follow-up of patient safety events or potential outbreaks. Staffing or workload requirements for ongoing compliance evaluation may also be a barrier. Nevertheless, efforts should be made to conduct regular evaluations as studies have observed reduced CLABSI rates when bundle compliance was strictly evaluated.^2^

There are several limitations to our study. Though CNISP represents 35% of all acute care beds in Canada, findings from this study may not be generalizable to all Canadian hospitals.

This survey was only able to assess the adoption of a CLABSI prevention bundle program as an infection prevention and control practice or policy in hospital ICUs and not the confirmed uptake from the date of program adoption. Bundle compliance reporting was low and limited to compliance at the time of the survey. Future studies will consider prospective study designs to accurately assess compliance with CLABSI bundle programs.

Survey respondent perceptions regarding hospital practices and patient safety culture may have may have introduced response bias. Not all participating hospitals reported rates across all years, so CLABSI rates could be skewed by smaller samples. Notably, only 1 hospital was included after 2016 for no bundle adopted in PICUs. Additionally, changes in infection prevention and control practices and public health measures and restrictions during the coronavirus disease 2019 pandemic could have affected CLABSI rates from 2020 onward.^10^ The derived IRRs were not controlled for hospital-related factors such as hospital size, teaching status, region, and temporal differences. Future research should further explore the association between bundle adoption (including individual components) and CLABSI rates in NICUs and adult mixed ICUs.

This study provides important insight into the landscape of CLABSI prevention bundles in CNISP hospitals across Canada, filling a gap in literature not previously explored. Most participating hospitals have adopted a CLABSI bundle program, with the extent of adoption and compliance varying by site and ICU type. CLABSI rates were lower in adult mixed ICUs and NICUs that had adopted a CLABSI insertion and maintenance bundle program compared to sites that did not.

## Supporting information

Zhou et al. supplementary material Zhou et al. supplementary material

## Supplementary material

The supplementary material for this article can be found at https://doi.org/10.1017/ice.2024.189.
