# Supplementary material for: The adoption and compliance to central line-associated bloodstream infection insertion and maintenance bundle programs in intensive care unit settings across Canada
Source: Infect Control Hosp Epidemiol. 2024 Dec 19;46(2):193–6. doi: 10.1017/ice.2024.189 (PMC11790321; doi:10.1017/ice.2024.189)
Supplement: Zhou et al. supplementary material [file S0899823X24001892sup001.docx]

**Supplemental Table.** Characteristics of Canadian acute care hospitals by self-reported adoption status of the SPS or CPSI CLABSI prevention bundle in intensive care unit settings.

| **Characteristic** | **Overall (N=46)** | **Adopted bundle, Yes (n=31)** | **Adopted bundle, No (n=15)** | ***p**** |
| --- | --- | --- | --- | --- |
| **Regional** | | | |  |
| Western | 15 (33%) | 13/31 (42%) | 2/15 (13%) | **0.007** |
| Central | 22 (48%) | 17/31 (55%) | 5/15 (33%) |  |
| Eastern | 8 (17%) | 1/31 (3.2%) | 7/15 (47%) |  |
| Northern | 1 (2%) | 0/31 (0%) | 1/15 (6.7%) |  |
| **Hospital Type** | | | |  |
| Adult | 26 (57%) | 19/31 (61%) | 7/15 (47%) | 0.403 |
| Mixed | 10 (22%) | 4/31 (13%) | 6/15 (40%) |  |
| Pediatric | 10 (22%) | 8/31 (26%) | 2/15 (13%) |  |
| **Bed Size Category** | | | |  |
| Small (1-200 beds) | 15 (33%) | 7/31 (19%) | 8/15 (53%) | **0.041** |
| Medium (201-499 beds) | 22 (48%) | 16/31 (52%) | 6/15 (40%) |  |
| Large (500+ beds) | 9 (20%) | 8/31 (19%) | 1/15 (6.7%) |  |
| **Teaching Hospital Status** | | | |  |
| Yes | 41 (89%) | 31/31 (100%) | 10/15 (67%) | **0.001** |
| No | 5 (11%) | 0/31 (0%) | 5/15 (33%) |  |

**Abbreviations:** CPSI, Canadian Patient Safety Institute; SPS, Solutions for Patient Safety.
**Note:** Percentage values are rounded to the nearest whole number.
*Pearson’s Chi-squared test.
